# Using Environmental DNA to Census Marine Fishes in a Large Mesocosm

**DOI:** 10.1371/journal.pone.0086175

**Published:** 2014-01-15

**Authors:** Ryan P. Kelly, Jesse A. Port, Kevan M. Yamahara, Larry B. Crowder

**Affiliations:** 1 Center for Ocean Solutions, Woods Institute for the Environment, Stanford University, Palo Alto, California, United States of America; 2 School of Marine and Environmental Affairs, University of Washington, Seattle, Washington, United States of America; University of California- Santa Barbara, United States of America

## Abstract

The ocean is a soup of its resident species' genetic material, cast off in the forms of metabolic waste, shed skin cells, or damaged tissue. Sampling this environmental DNA (eDNA) is a potentially powerful means of assessing whole biological communities, a significant advance over the manual methods of environmental sampling that have historically dominated marine ecology and related fields. Here, we estimate the vertebrate fauna in a 4.5-million-liter mesocosm aquarium tank at the Monterey Bay Aquarium of known species composition by sequencing the eDNA from its constituent seawater. We find that it is generally possible to detect mitochondrial DNA of bony fishes sufficient to identify organisms to taxonomic family- or genus-level using a 106 bp fragment of the 12S ribosomal gene. Within bony fishes, we observe a low false-negative detection rate, although we did not detect the cartilaginous fishes or sea turtles present with this fragment. We find that the rank abundance of recovered eDNA sequences correlates with the abundance of corresponding species' biomass in the mesocosm, but the data in hand do not allow us to develop a quantitative relationship between biomass and eDNA abundance. Finally, we find a low false-positive rate for detection of exogenous eDNA, and we were able to diagnose non-native species' tissue in the food used to maintain the mesocosm, underscoring the sensitivity of eDNA as a technique for community-level ecological surveys. We conclude that eDNA has substantial potential to become a core tool for environmental monitoring, but that a variety of challenges remain before reliable quantitative assessments of ecological communities in the field become possible.

## Introduction

A key component of understanding marine ecosystems, and of implementing science-based policy in those ecosystems, is the development of comprehensive environmental monitoring programs. Important attributes of such programs include the ability to assess biodiversity and track the status of indicator species [1]. Examples of current marine monitoring programs along the west coast of the United States include the Partnership for Interdisciplinary Studies of Coastal Oceans (PISCO; an academic collaboration), California Cooperative Oceanic Fisheries Investigations (CalCOFI; a public-private partnership), the nonprofit Reef Check, and programs affiliated with NOAA Fisheries (a federal agency), among others. These provide data on species diversity and community composition using visual surveys, trawls, seines and tissue biopsies. While they are critical sources of data, such monitoring techniques can be expensive, time-consuming, invasive and prone to high false-negative detection rates [2]–[4]. More efficient, more cost-effective, and more sensitive methods are thus desirable for ecosystem assessments as well as for improving baseline ecological knowledge about marine ecosystems.

Advances in DNA sequencing technology and bioinformatics have significant potential to strengthen biological monitoring in the ocean. All living things contain DNA and generate waste (e.g., sloughed cells, metabolic waste) that persists in the environment for some period of time. Sampling this environmental DNA (eDNA) could be a powerful means of surveying large portions of the living environment and would offer increased resolution and accuracy when compared to manual methods for environmental sampling [5]. High-throughput sequencing of microbial eDNA is a well-established approach used by microbiologists that has uncovered vast diversity in a wide variety of environments [6]–[9]. Only recently has similar work on eukaryotes come to the fore, and the last two years have seen an explosion of interest in relating selectively amplified animal DNA to the distribution and abundance of species in the field [10]–[15].

Freshwater habitats, and to a lesser extent marine systems, have been the focus of many pioneering eDNA studies [5], [11], [16]–[20]. This work has shown that it is possible to amplify and sequence DNA from environmental water samples to detect individual animal species of interest even when target species are present at very low abundances—such as in the case of rare and endangered species [2], [20]. Furthermore, lab-based work has demonstrated a quantitative relationship between density or biomass of a species and amount of DNA present in the species' habitat [20], and limited field-based surveys are consistent with this finding [17], [21]. Moving from presence/absence surveys toward field methods that can establish the relative abundance or actual quantification of individuals within species—and from species-specific surveys towards community assessments—would provide a broadly practical tool for environmental monitoring.

Here, using a 4.5×10^6^-L tank at the Monterey Bay Aquarium (Monterey, California, USA) as a mesocosm, we test the feasibility of eDNA sequencing for reconstructing the known community composition of the tank which is composed of 12 species, including bony and cartilaginous fishes and sea turtles. To do so we use a single set of vertebrate-specific mitochondrial DNA (mtDNA) primers that require no previous knowledge of target species. Furthermore, we use the Illumina MiSeq platform to obtain increased sequencing depths relative to 454 pyrosequencing, the mainstay of eDNA studies to this point. Using a known mesocosm community—which varies by four orders of magnitude in number of individuals per species and over a similar range in biomass per species—allows us to assess the accuracy of the technique by establishing false detection rates. We find a taxonomic bias towards bony fishes, and that within amplified bony fish species, rank DNA sequence abundance correlates with rank biomass abundance, suggesting that concentration of eDNA is in part a function of species' abundance. The relationship between proportion of sequences recovered and relative biomass present is strongly nonlinear, but the data in hand do not allow us to develop a quantitative relationship between the two. We close by addressing some of the methodological challenges associated with moving from presence/absence detection to abundance of identified groups of marine vertebrates, and the importance of these challenges for marine monitoring and marine ecology generally.

## Materials and Methods

### Ethics Statement

This study was approved by the Monterey Bay Aquarium.

### Mesocosm Sampling

We collected seawater samples in February 2013 from the 4.5×10^6^-L Open Sea Tank at the Monterey Bay Aquarium. The Open Sea Tank is inhabited by a selection of species from the Pacific Ocean: two species of tuna (*Thunnus orientalis* and *Thunnus albacares*), an ocean sunfish (*Mola mola*), a school of sardines (*Sardinops spp.*), dolphinfish or mahi-mahi (*Coryphaena* sp.), and others (Table 1). The seawater is re-circulated, with turnover approximately every 2 hours. Seawater entering the Aquarium is pumped directly from Monterey Bay via an intake pipe extending 350 m into the bay. The water then passes through other aquarium exhibits and several filters before entering the Open Sea Tank. We took a 20-L sample from the intake pipe just prior to its discharge point into the tank (“intake sample”), and another 20-L sample of water from the tank itself at surface level (“tank sample”). We divided each sample into one 15-L and five 1-L samples for analysis; the results presented below focus principally on sequences derived from these 1-L subsamples.

**Table 1:** Species composition of the Monterey Bay Aquarium Open Sea Tank and presence/absence based on eDNA detection.

Species	Common name	Family	Approx.count	Estimated biomass in tank (kg)	Lowest taxonomic rank detected with eDNA
**Bony fish**					
*Coryphaena hippurus*	Dolphinfish	Coryphaenidae	6	84	Genus
*Mola mola*	Ocean sunfish	Molidae	1	1,000	No detect
*Naucrates doctor*	Pilot fish	Carangidae	17	12.75	Family
*Sarda chiliensis*	Pacific bonito	Scombridae	15	45	Family
*Sardinops sagax*	Sardine	Clupeidae	13,000	2,600	Genus
*Scomber japonicus*	Chub mackerel	Scombridae	17	7.7	Genus
*Thunnus albacares*	Yellowfin tuna	Scombridae	11	748	Genus
*Thunnus orientalis*	Pacific bluefin tuna	Scombridae	7	1,890	Genus
**Cartilaginous fish**					
*Carcharhinus plumbeus*	Sandbar shark	Carcharhinidae	1	65	No detect
*Dasyatis violacea*	Pelagic stingray	Dasyatidae	2	5	No detect
*Sphyrna lewini*	Hammerhead shark	Sphyrnidae	2	109	No detect
**Turtle**					
*Chelonia mydas*	Green sea turtle	Cheloniidae	2	258	No detect

Samples were vacuum-filtered onto 0.22-µm Durapore membrane filters (Millipore, MA, USA). Filters were then folded inwards, placed in 2 ml tubes and stored at −80°C until DNA extraction, which took place within 48 hours.

In addition, we collected two samples of the commercial feed dispersed daily into the Open Sea Tank. These included an aquatic gel diet (“gel sample”) and sinking pellet feed (“pellet sample”) (Mazuri, USA). Food samples were stored at −20°C until DNA extraction.

### DNA extraction

DNA was extracted from the two water sample filters using the DNeasy Blood and Tissue Kit (Qiagen, USA). Manufacturer's protocols were used during all steps except 2× volumes of buffer ATL, proteinase K, buffer AL and ethanol were used during lysis. DNA from the two feed sample filters was extracted using the PowerSoil DNA Isolation Kit (MoBio laboratories, CA, USA) and a preliminary bead-beating step. For bead-beating, 0.25 g of feed were added to a 0.1 mm PowerBead tube (MoBio laboratories, CA, USA) containing 60 µl of Solution C1 and then shaken in a FastPrep-24 (MP Biomedicals, USA) at 6.5 m/s for 60 sec. DNA for the water and feed samples was eluted in a final volume of 100 and 40 µl respectively. eDNA concentrations were determined using the Qubit dsDNA HS Assay (Invitrogen, CA, USA).

### PCR amplification

We amplified target samples using PCR primers designed by Riaz and coauthors to amplify vertebrate-specific fragments from the mitochondrial 12S rRNA gene [22]. A 106 bp fragment from a variable region of the 12S rRNA gene was amplified with the primers F1 (5′-ACTGGGATTAGATACCCC-3′) and R1 (5′- TAGAACAGGCTCCTCTAG-3′). We initially validated the primers on tissue samples for species known to inhabit the Open Sea Tank, including yellowfin tuna (*Thunnus albacares*) and dolphinfish (*Coryphaena hippurus*) (data not shown). After initial validations, we amplified water samples as follows: each 25 µl PCR reaction contained 5 µl DNA extract, 12.5 µl HotStarTaq Plus Master Mix (Qiagen, CA, USA), 1 µl of each primer (10 µM) and 5.5 µl ddH_2_O. PCR conditions consisted of an initial incubation at 95°C for 5 minutes followed by 35 cycles of 95°C for 15 seconds, 57°C for 30 seconds, and 72°C for 30 seconds. To combat stochasticity in PCR results, we carried out five individual PCR reactions per sample and then pooled amplicons, with the exception of the three technical replicates (1-L tank samples) whose individual PCR products were sequenced separately. Fragment size was verified on 2.5% agarose gels stained with ethidium bromide and PCR products were purified using a MinElute PCR purification kit (Qiagen, CA, USA).

Following our initial results, we also used species-specific primers to test for the presence of Green sea turtle (*Chelonia mydas*) in the tank, using the primers LTCM2 (5-CGGTCCCCAAAACCGGAATCCTAT-3) and HDCM2 (5-GCAAGTAAAACTACCGTATGCCAGGTTA-3) [23] which target the mtDNA control region. Amplification followed the protocol above, but used a 60°C annealing temperature. We designed a synthetic plasmid containing a 666 bp sequence from the mtDNA control region of *C. mydas* to serve as a positive control for the PCRs. PCR products were purified using the MinElute PCR purification kit and sequenced with an ABI 3730×l sequencer (Elim Biopharmaceuticals, Inc., Hayward, CA). The resulting sequences were trimmed using Geneious Pro v6.0.5 (Biomatters Ltd.) and compared against the National Center for Biotechnology Information (NCBI) nonredundant nucleotide database. Sequences were submitted to GenBank (accessions KF891283 and KF891284).

### Next-Generation DNA Sequencing

Illumina library construction and sequencing were performed at the Genome Sequencing and Analysis Core Resource at Duke University. Indexed amplicon libraries were constructed using a KAPA Library Preparation Kit (KAPA Biosystems, USA). Libraries were pooled in equimolar concentrations and multiplex sequenced (150 bp paired-end) on a single flowcell using the Illumina MiSeq. To improve the data quality of low-diversity samples (i.e., single-fragment PCR products such as ours), the run included a 30–50% PhiX DNA spike-in control. Raw reads are available by sample in the NCBI Sequence Read Archive (SRA; accessions SRX375380-375387).

### Bionformatic analyses

All bioinformatic analyses were implemented using the R statistical package version 3.0.1 (R Core Development Team 2013) to perform analyses with the Bioconductor package ShortRead [24] and with external unix-based programs QIIME version 1.6.0 [25], FLASH v.1.0.3 (http://ccb.jhu.edu/software/FLASH/) and MEGAN [26].

Initial quality filtering of reads was performed using QIIME. Forward and reverse primer sequences were removed, allowing for three mismatches in the primer sequence. Low-quality bases were removed by trimming reads at the beginning of the first poor quality window, defined as a 10 bp region with an average quality score less than 25. Reads with more than 5 ambiguous bases or a 6 bp homopolymer run were also omitted. Reads meeting these criteria were further filtered for minimum (55 bp) and maximum (106 bp) lengths. Only those paired-end reads meeting the filtering criteria in both the forward and reverse directions were included for further analysis.

Paired-end reads were aligned and merged using FLASH with a minimum overlap of 15 bp and a 10% error rate (i.e., allowing 1 mismatch). The merged sequences were sorted in order of decreased abundance and then clustered using QIIME into operational taxonomic units (OTUs), with taxonomic identities assigned using a custom vertebrate mtDNA reference database. The custom database was generated by downloading all complete vertebrate mitochondrial genomes from GenBank and then randomly selecting one species per genus to remain in the database, yielding a database with a total of 1,552 complete mitochondrial genomes. We then supplemented this database with full or partial 12S sequences for three species known to be in the Open Sea Tank but lacking complete mitochondrial genomes in GenBank: *Coryphaena hippurus*, *Sarda sarda* and *Sphyrna lewini*. One genus, *Naucrates*, lacked a 12S fragment in GenBank and therefore any sequences recovered corresponding to the *Naucrates ductor* individual in the tank could not be assigned to the species or genus level.

Reads were clustered against the reference database at ≥97% similarity using UCLUST [27] implemented within QIIME. A 97% identity threshold allows for taxonomic assignment to at least the family level for the known taxa in the tank using the vertebrate-specific 12S mtDNA fragment. Tank taxa were assigned to the genus level only if their 12S mtDNA fragment was specific to a single genus at the ≥99% similarity level. Only genera accounting for greater than 0.01% of reads in a sample were retained, a conservative means of accounting for unique sequences generated via sequencing error.

We visualized taxonomic assignments using MEGAN, and collapsed to the levels of taxonomic genus or family as appropriate for analysis using the NCBI taxonomy implemented in MEGAN. Rarefaction curves were generated in R using as a representative dataset one of the three 1-L tank samples (23 unique genera, 843,746 assigned sequences). The dataset was subsampled 10^5^ times, varying the sizes of the subsamples between 1 and 8×10^5^ sequences. The Shannon-Weiner diversity index [28] was calculated with the equation H′ = Σ*p_i_*ln*(*p_i_*), where *p_i_* is the proportion of sequence reads that are classified into the *i*-th taxonomic family.

### Mixing model

We developed a mixing model to estimate the contributions of four different sources of DNA in the sampled aquarium tank: intake water, two different commercial feed sources (gel and pellet forms) and endogenously generated DNA shed by the species in the tank itself. We sequenced 12S mtDNA from the intake, gel feed, and pellet feed—in addition to the tank samples themselves—and used the model to parse these sources and thereby infer the abundances of DNA shed by the tank species (Eq. 1). This model assumes that there are no additional sources of DNA to the Open Sea Tank, and uses the recovered sequence proportions for all assigned taxa (genus-level, 99% identity) as the dataset used to infer the unknown parameters.
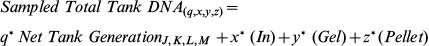
(Eq. 1)Where:

Sampled Total Tank DNA = A vector reflecting the distribution of recovered DNA sequences from the tank samples among all taxa detected (expressed as proportions, such that the vector sums to 1)

Net Tank-Generation_I,K,L,M_ = Estimated net DNA sequence generation from tank taxa I, K, L, M

In = A vector reflecting the distribution of recovered DNA sequences from the intake sample among all taxa detected (expressed as proportions, such that the vector sums to 1)

Gel = A vector reflecting the distribution of recovered DNA sequences from the sample of gel feed among all taxa detected (expressed as proportions, such that the vector sums to 1)

Pellet = A vector reflecting the distribution of recovered DNA sequences from the sample of pellet feed among all taxa detected (expressed as proportions, such that the vector sums to 1)

*q* = The estimated proportion of overall recovered tank DNA generated by amplified taxa occurring in the tank

*x* = The estimated proportion of overall recovered tank DNA due to the tank intake

*y* = The estimated proportion of overall recovered tank DNA due to the gel feed

*z* = The estimated proportion of overall recovered tank DNA due to the pellet feed

J,K,L,M = The proportions of DNA generated in the tank due to each of the four tank taxa detected (*Coryphaena*, *Scomber*, *Sardinops*, and *Thunnus*, respectively) with the 12S mtDNA markers.

We then randomly sampled 5×10^6^ sets of values for the eight unknowns (*q*, *x*, *y*, *z*, J, K, L, M) drawn from two independent Dirichlet distributions such that the sets of proportions (q, x, y, z) and (J, K, L, M) each summed to 1, and fit a linear model (following Eq. 1) to the data given the sampled parameters. We optimized the model by minimizing the residual sum of squares. The overall best model (R^2^ = 0.985) and density distributions for each parameter estimate are provided in the results section. The model was robust to a variety of constrained parameter values (Figures S1 and S2; Text S1). All calculations were implemented in R 3.0.1.

## Results

We generated a total of eight sets of 12S mtDNA sequences: tank intake (1-L and 15-L samples), two forms of tank feed (gel and pellet forms), three technical replicates of tank surface water (derived from the same 1-L subsample), and one 15-L tank surface water sample. Illumina MiSeq analysis yielded an average of 1.007 million reads per multiplexed sample after quality filtering and pairing of bidirectional reads (74–80% sequence recovery; see Table 2). The pre-processed reads were of high quality, with over 90% of the paired-end reads per sample having a phred score >Q30 (Figure S3).

**Table 2:** Summary statistics for the Illumina MiSeq run.

	Collection Date	Location	Water volume (L) or Mass (g)	No. of reads sequenced	No. of reads passing QC	No. of OTUs^d^	Shannon diversity index (H′)
**Sample 1**	2/27/13	Tank intake	1.0^a^	1,130,048	875,244 (77.5%)	28	1.12
**Sample 2**	2/27/13	Tank intake	15.0^a^	1,293,887	1,012,509 (78.3%)	31	1.08
**Sample 3**	2/27/13	Tank surface	1.0^b^^,^ c	1,233,529	997,294 (80.8%)	27	1.33
**Sample 4**	2/27/13	Tank surface	1.0^b^^,^ c	1,262,477	1,009,030 (79.9%)	30	1.33
**Sample 5**	2/27/13	Tank surface	1.0^b^^,^ c	1,316,108	1,043,136 (79.3%)	22	1.32
**Sample 6**	2/27/13	Tank surface	15.0^b^	1,510,565	1,172,724 (77.6%)	22	1.17
**Sample 7**	2/27/13	Feed (gel diet)	0.25 g	1,240,934	920,941 (74.2%)	35	0.80
**Sample 8**	2/27/13	Feed (pellet)	0.25 g	1,298,775	1,028,869 (79.2%)	35	0.52

^a^ Sourced from same 20.0-litre sample.

^b^ Sourced from same 20.0-litre sample.

^c^ Sourced from same DNA extract.

^d^ Refers to number of OTUs matching the vertebrate reference dataset.

mtDNA = mitochondrial DNA; OTU = operational taxonomic unit.

We detected no substantial differences among the three sequencing replicates that were sourced from the same 1-L water sample. The relative abundances for each taxon detected were very similar across the triplicates, and only two rare taxa (*Meleagris* and *Oncorhynchus*), were unique to just one sample (Figure S4).

The larger sampling volumes (15-L) for the intake and tank samples had lower diversity than the 1-L samples as evidenced by the Shannon diversity indices and taxonomic profiles (Table 2; Figure S4). For example, *Enhydra* and *Canidae* were present in low concentrations in the 1-L tank samples but absent in the 15-L sample (Figure S4).

**Figure 1:**
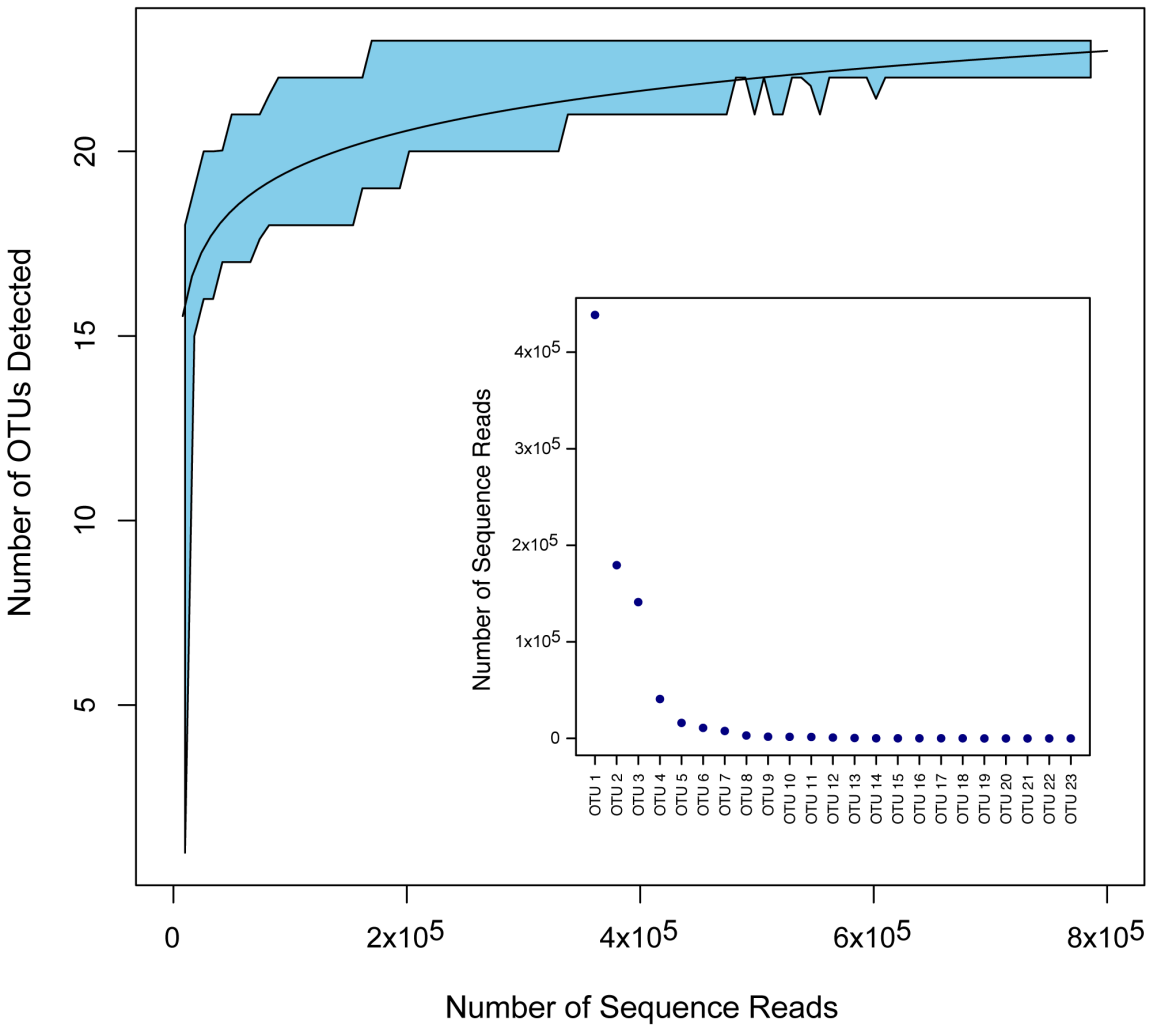
Rarefaction curve for 10^5^ subsamples of OTU clusters recovered from the mesocosm Aquarium tank. The best-fit curve is shown for clusters of ≥99% identity. 95% confidence interval shown as shaded area, derived from a sliding window analysis of the subsampled data, using a window size of 20,000 reads and 20% overlap between windows. The frequency distribution of assigned reads recovered is shown in the inset figure.

Rarefaction analysis revealed our sequencing depth to be sufficient to identify all or nearly all of the amplified taxa present in the 1-L tank (Figure 1; genus-level assignments for 23 taxa assigned at 99% identity). Genera with few occurrences (on the order of <0.01% of sequences recovered in the present dataset; *e.g. Meleagris*) can reliably be detected with a depth of ca. 200,000 reads.

### Community presence/absence

The 12S fragment was sufficiently variable to discern vertebrates at the level of taxonomic family, and in some cases could distinguish taxa at the genus level. We recovered 12S sequences from 4 out of the 5 bony fish families present in the tank exhibit (Table 1). Molidae DNA—a family with a single representative in the tank, *Mola mola*—was not present in either the 1-L or 15-L tank samples, although it had been detected in an earlier, preliminary round of sequencing using the same primer set.

Within those bony fish families detected, the 12S fragment could resolve, and did detect, 4 of 8 genera present in the mesocosm tank (*Coryphaena*, *Sardinops*, *Scomber* and *Thunnus*). One genus, *Naucrates* (pilot fish) could not have been detected and assigned as there is no 12 s sequence data available for this genus in GenBank; similarly, *Sarda* was indistinguishable from *Thunnus* at the 99% identity threshold.

We recovered no sequences attributable to the other 4 taxonomic families represented by species present in the mesocosm tank, which included three cartilaginous fishes and one sea turtle (Table 1). In sum, we detected sequences corresponding to 4 out of the 9 taxonomic families present in the tank, with a significant bias in favor of bony fishes. This is a false negative rate of 5/9 (0.55) for all vertebrate families, and of 1/5 (0.2) for bony fish families.

To determine if green sea turtle DNA was in fact absent from the water samples, we probed the 1-L tank sample and an additional 1-L Open Sea Tank sample collected the previous year (Oct. 2012) with PCR primers targeting the mtDNA control region of green sea turtle. These samples successfully amplified the intended target, which we verified with Sanger sequencing (Figure S5).

### Relative abundance

The mesocosm Open Sea Tank contained a mixture of DNA from four sources: the intake water, two different commercial feed sources (gel diet and pellet forms) and the target DNA shed by the species in the tank itself. We therefore used a mixing model—based upon the proportion of recovered sequences from each identifiable source—to estimate the contributions of these four different sources of DNA in the sampled aquarium, and estimated the relative abundance of tank-species-generated DNA sequences after subtracting out the sequences derived from other sources.

**Figure 2:**
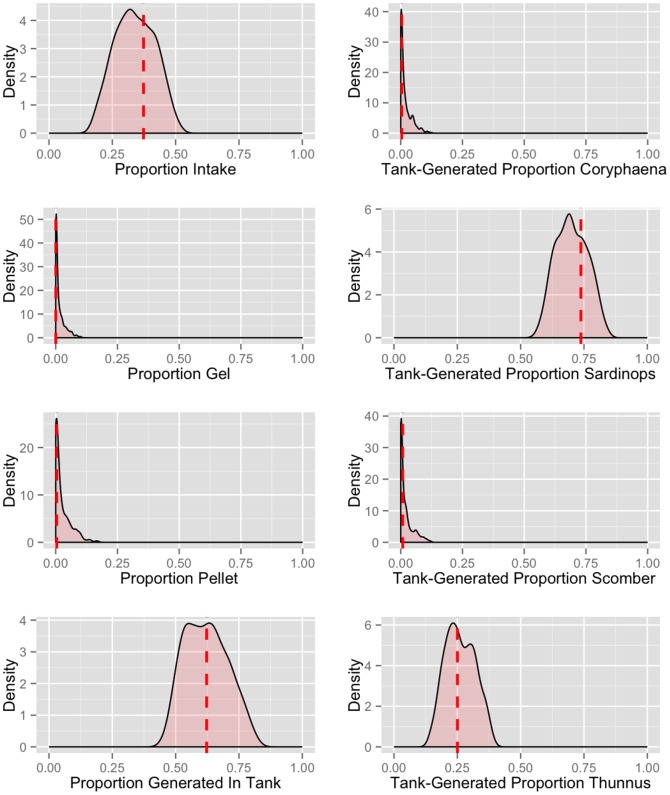
Density distributions for sampled parameter values in the mixing model. Shaded areas represent 5×10^4^ model results constituting the 99th percentile of goodness-of-fit. Dashed red lines indicate the parameter value for the overall best-fit model described in the main text. Note that the optimal set of parameter values does not necessarily coincide with the point of maximum density for any given parameter, as the parameters are not independent of one another. Parameters in the left-hand column represent proportions of each source of DNA mixed into the sampled aquarium tank; parameters in the right-hand column represent proportions of DNA generated by each genus whose 12S mtDNA was detected within the tank.

The best-fit model (See Text S1) explained 98.9% of the variance in the observed tank sequence proportions. Best-fit model parameters suggest over two-thirds (69%) of the overall mesocosm DNA is generated by species living in the tank (50.2–83.5%; 95% CI based on 5×10^4^ best-fit models), with the contributions of the intake, gel diet feed, pellet feed being 30.9% (15–45%), 0.0014 (3.2×10^−5^–8.2%), and 0.024% (2.7×10^−5^–9.8%) respectively (Figure 2).

**Figure 3:**
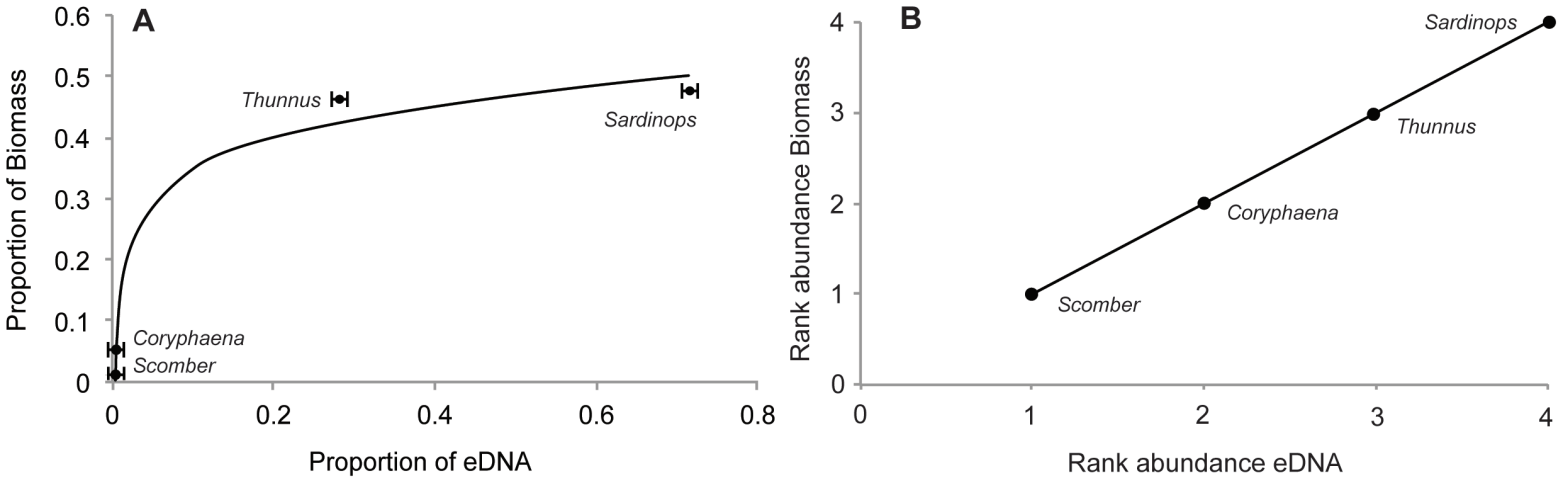
Comparison of the proportion of eDNA sequences recovered to estimated species biomass in the 1-L tank sample. (A) Relationship between the proportion of eDNA sequences and proportion of biomass in the tank (Best fit line = y = 0.0759*In(x)+0.5257) and (B) the rank abundances of these proportions for the four tank exhibit genera detected. The error bars represent the standard deviation of the three individual PCR replicates for the 1-L tank sample.

Under this set of model parameters, *Sardinops* and *Thunnus* accounted for the largest percentage of the eDNA sequences in the 1-L tank sample (71.4% (59.7–81.5%) and 28.2% (16.6–36.6%), respectively) (Figure S2). *Coryphaena* and *Scomber* made up the remaining small percentage of reads generated in the tank (0.128% and 0.00145%, respectively). These modeled proportions of in-tank eDNA generation show a nonlinear relationship with the known biomass of species in the mesocosm tank (Figure 3A), and the rank abundance of modeled eDNA generation matches the rank abundance of biomass exactly (Figure 3B).

### eDNA from non-tank species

**Figure 4:**
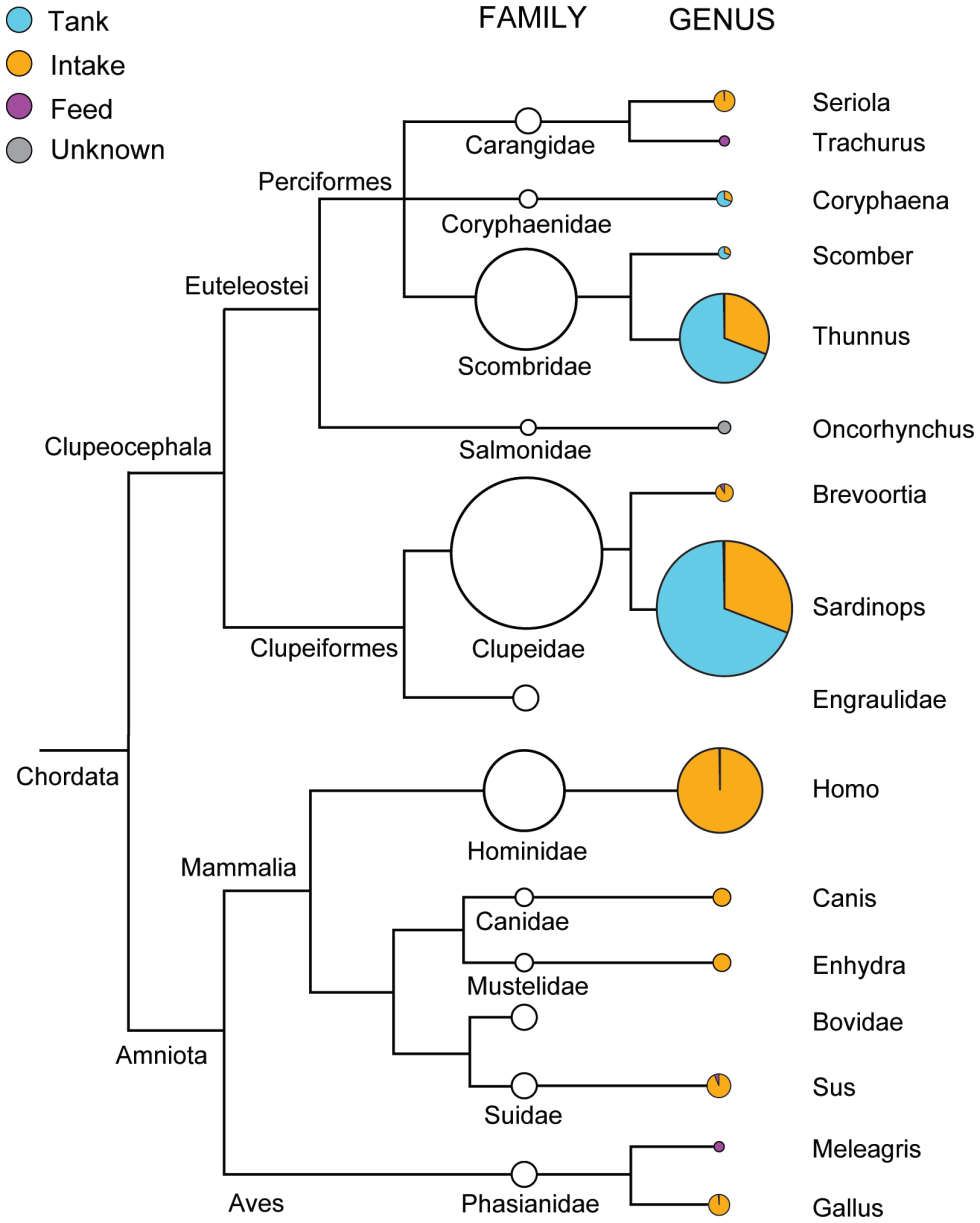
Relative abundances and sources of the taxa detected in the 1-L tank sample. Abundances are based on the weighted average of the three sequencing replicates of 1-L tank samples. The size of the circles is proportional to the number of sequence reads, with the largest circle equivalent to 425,178 reads (50.6% of the tank sample). Tank (blue) refers to DNA that was generated within the tank. Feed (purple) includes both gel and pellet diets. Unknown (gray) refers to DNA that was detected in the tank but was not generated in the tank and did not appear in the intake or feed. The tree diagram is derived from NCBI taxonomic groupings, rather than from an evolutionary phylogenetic analysis.

Approximately 25.5% of the tank sequences (with clusters of ≥97% sequence identity collapsed to the level of taxonomic family) were assigned to taxa not living in the mesocosm tank (Figure 4; Figure S4). These exogenous sequences were attributable to the intake and feed sources, and included an array of other bony fish, mammals, and birds. Human DNA from the intake accounted for the majority of this exogenous DNA (62.0%). Common vertebrate species—Bovidae (cow), Suidae (pig), Phasianidae (chicken and turkey) and Engraulidae (anchovy)—composed 29.2% of the remaining non-tank species DNA. Sea otter (*Enhydra*), which are common in the nearshore Monterey Bay environment from which the intake water is drawn, was also present in both the 1-L intake and tank samples. Commercial feed accounted for only a small proportion of the tank DNA, and consisted mainly of menhaden (*Brevoortia*) (Figure S4). Except for a small number of sequences the genus *Oncorhynchus* (family Salmonidae), all DNA detected in the tank could be sourced back to either the intake, feed or endogenous production within the tank, resulting in a false positive rate of 1/12 (8.3%) for exogenous DNA at the level of taxonomic family, or 0.07% of recovered sequences corresponding to a taxon not present elsewhere in the study.

## Discussion

### Assessing community membership with a common mtDNA fragment

Our goal was to assess the vertebrate community present in a mesocosm by amplifying and sequencing the same fragment of mtDNA from a variety of vertebrates, building on earlier work by Thomsen and colleagues [5], [20]. Surveying a community of known composition and biomass allowed us to evaluate the error rates of the eDNA sequencing technique, and—after subtracting out the DNA contributions from exogenous sources—to compare the proportion of taxon-specific sequences with the corresponding proportional biomass of species in the tank.

We detected the majority of the bony fish taxa present in a 1-L sample of a 4.5×10^6^-L mesocosm tank by sampling eDNA amplified with vertebrate-specific mtDNA primers. Cartilaginous fishes and a sea turtle—which were also present in the tank during sampling—were not detected using eDNA sequencing. However, using species-specific primers for *C. mydas*, turtle DNA was found to be present in the aquarium samples. One bony fish, *Mola mola*, that was present in the tank was not recovered at levels sufficient for analysis, although previous results from a pilot study (data not shown) suggest this species does amplify and sequence with this same 12S primer set.

Consistent with previous findings in fungal communities [29], our results suggest that surveying a community of moderate species diversity using eDNA requires a degree of caution due to biases in detection. Sequencing depth, differential DNA shedding rates and/or preferential amplification of species may be important considerations when interpreting community composition eDNA profiles as has been noted elsewhere [30], [31]. Due to limitations in sequencing depth, especially when multiplexing, rarer organisms are less likely to be detected. Organisms may be less abundant because they are simply less prevalent in the given environment or because they shed less DNA. Uneven species abundances can lead to lower apparent species richness as a result of more common species swamping out signals from rarer amplicons [32]. This effect is likely exacerbated during PCR, in which more common templates will be preferentially amplified.

Primer bias may also explain the sequence detection and abundance results. Ideally it would have been beneficial to test whether the 12S vertebrate primer set amplifies sea turtle, hammerhead or sandbar shark DNA from a complex matrix such as the Open Sea Tank, but tissue samples for these species were not available. However, based on analysis of the number of base pair mismatches in the 12S vertebrate primers, it is likely these primers preferentially amplify bony fishes (Figure S6). The 12S rRNA sequences for the most abundant species in the tank (sardine and tuna) had no mismatches to the vertebrate primer set. On the other hand, the cartilaginous fishes and sea turtle had two mismatches in the forward primer region. *Coryphaena* and *Scomber* each had one mismatch in the reverse primer region, which may explain their decreased abundance relative to the other bony fishes. Despite these potential sources of bias, detecting the majority of the bony fish taxa present in a large mesocosm by sequencing a single fragment of mtDNA is a substantial step toward making eDNA surveys a useful tool for monitoring in the field, and for assessing some of the limits of genetic monitoring methods.

### Assessing the contribution of different sources of DNA to the mesocosm

We estimated that over two-thirds (69%) of the amplified 12S mtDNA in the Aquarium tank was generated by the animals in the tank, given the best-fit model of genus-level sequence abundances from each DNA source. The background intake water appears responsible for much of the remainder (30.9%, CI 14.9–45.4%), with the two types of feed accounting for only fractions of a percent of total tank DNA.

A weakness of the mixing model is that it assumes a linear relationship between template DNA present and proportion of sequences generated. However, even in the absence of a linear relationship, it is clear that the major sources of DNA in the tank are the endogenously-generated tank-species DNA and the background signal from the intake, rather than from the feed. For example, although *Brevoortia* (Atlantic menhaden, a common ingredient in commercial feed) constituted 69.6% and 46.2% of sequences recovered from gel and pellet feeds, respectively, that genus made up only 0.185% of the sequences recovered from the tank sample. Similarly, turkey (*Meleagris*) accounted for 18.6% of the gel feed, but only 0.0069% of the sequences in the tank sample. Similar evidence points to the intake as constituting a minority of DNA: while human (*Homo*) DNA dominated the intake sample (50.7% of all sequences), human sequences were only 16.9% of those recovered from the tank. Even in the absence of a linear relationship between template and sequence, then, it is very likely that endogenously-generated DNA is a majority of the tank signal.

While the model also estimated only a small proportion of the tank DNA as being sourced from the commercial feed, it appears to have underestimated the feed contribution of sequences from the most abundant feed species (e.g. *Brevoortia* and *Meleagris*). Atlantic *Brevoortia* (menhaden) is the main component of both feeds (Figure S4), and is unlikely to be a naturally occurring source of DNA in water from Monterey Bay or in the mesocosm tank. It is therefore likely that all or nearly all of the *Brevoortia* DNA recovered from the tank derives from the commercial feed. Because the best-fit model represents source contributions that integrate over all detected taxa in the sources and the tank, the modeled proportion of *Brevoortia* from feed appears not to reflect the most likely source of DNA for this particular taxon.

We note also that the presence of sea otter (*Enhydra*) in both the 1-L intake and tank samples is evidence that this 12 s mtDNA primer set may be useful for monitoring marine mammals in the Monterey Bay area. In addition, the human and domesticated animal (e.g. pig, chicken, cow) signals may in part be attributed to contamination of commercial PCR reagents [33] or to actual presence in the environment. These are commonly detected in environmental DNA samples, and may be addressed in part by designing specialized primers to block their amplification [34].

### From presence/absence to relative abundance

Of the tank-generated DNA, the vast majority appears to have been generated by the two most abundant taxa in the tank: sardines (72%) and tuna (27.8%), with the less abundant *Coryphaena* and *Scomber* accounting for the small remainder (0.14% and 0.0015%, respectively). To a first approximation, then, we observe two high-sequence-abundance taxa and two low-sequence-abundance taxa.

The relative abundance of sequences exactly preserves the relative abundance of bony fish biomass in the tank for the taxa amplified (Figure 4B). Our results indicate that the relationship between biomass and eDNA generated is nonlinear, consistent with other eDNA results, within species [20] and between prey species in scat [30], [35]. However with the present dataset we are unable to discern whether this nonlinear relationship is due to 1) taxon-specific differences in amplification and sequencing, 2) the nonlinear nature of PCR, which exponentially increases amplicons and is subject to stochasticity even in the absence of taxonomic amplification bias, or 3) more abundant species actually shedding a disproportionate amount of DNA into the surrounding environment.

It seems likely that all three of these mechanisms play a role, and disentangling the importance of each will be necessary before community-level eDNA surveys are to be routinely useful in the field. Variables such as metabolism and surface area no doubt influence a species' shedding rate. Animals with higher metabolic needs are known to have higher rates of cell generation and division [36] which may lead to higher rates of DNA shedding. Surface area may prove to be an influential factor, and in this case schools of small fish would shed more genetic material than a single large fish of the same mass. Distinguishing between mass and number of individuals will be important, for example, for stock assessment or age-structure analyses integral to monitoring commercial fish stocks or marine protected areas.

The data in hand are therefore insufficient to develop a rigorous model that relates the abundance of individual species to the concentrations of corresponding eDNA present in an environmental sample. Nevertheless, we found that eDNA sequences do reflect the relative abundance of amplified taxa in the mesocosm, suggesting that eDNA is a function of species' abundance or of additional biological variables such as metabolism or surface area.

### Prospects for Community Surveys Using eDNA

Three things fundamentally constrain the ability to accurately identify and census organisms using eDNA: the variability of the sequenced DNA fragment among related species, the length of the sequenced fragment, and the completeness of the reference database [14], [15]. Context-specific constraints—such as the temporal and spatial variability of eDNA within the sampling area, may also limit the effectiveness of eDNA surveys in particular cases.

Our focal 106-bp fragment of 12S mtDNA is sufficiently variable that in some cases it can distinguish congeners—for example, the sardines *Sardinops sagax* and *S. melanostictus*—but only reliably distinguishes between vertebrate families. For cases in which taxa differ by one or a few base pairs in 100 bp—such as is likely the case for many congeneric species at 12S or similar mtDNA loci [37]—a longer fragment is desirable both to more confidently distinguish species and to unambiguously differentiate between random sequencing error and real genetic differences [38]. Screening multiple marker genes (e.g. COI, CytB, 12S, 16S or nuclear genes) would improve community level analyses by offering lower taxonomic resolution and by reducing primer bias and the false negative rate of detection. However, the majority of universal primers targeting vertebrates and other metazoans have been developed for use with tissue samples or gut contents as part of diet studies [39]–[42], and as a result non-specific amplification can occur when applied to mixed environmental samples. There is thus a need for primers designed for *in situ* monitoring of marine ecosystems.

In the case of vertebrate and other macrofaunal surveys, the completeness of the reference database is likely to be of less concern than in eDNA surveys of lesser-known microbial fauna. In the present study, Genbank contained the 12S fragment of interest for all but one of the vertebrate species in the mesocosm. This coverage is a substantial argument in favor of barcoding-like mtDNA surveys using familiar 12S, 16S, COI, or cytB regions [42], [43], and these regions are likely to be useful for many future eDNA applications.

A substantial source of uncertainty in eDNA studies is the degree to which spatial and temporal variability influence results. This is particularly the case in marine and aquatic environments, in which genetic material is expected to diffuse and be transported away from its source organisms. We designed our experiment to be a robust test of vertebrate-specific primers eDNA in a community of known composition, and we assumed that a controlled, well-mixed environment would exhibit less patchiness in eDNA detection and that therefore replication of a single time point would be sufficient to assess variability. Nevertheless, it is possible that DNA is spatially heterogeneous in the tank, and we would expect rare amplicons (the tail of the abundance distribution) to be especially sensitive to such heterogeneity. Thomsen and colleagues [5] have shown spatiotemporal variability to be a surmountable factor in the context of detecting rare species in lakes and streams, and as the price of DNA sequencing continues to drop, it will become feasible to sample more thoroughly over time and space to assess the variability of eDNA survey results.

## Conclusion

Marine ecology and environmental science are often limited by a lack of data and by the costs of collecting those data. More efficient, more cost-effective, and more sensitive methods could thus revolutionize ecosystem assessments and improve the way in which we collect baseline ecological data about marine ecosystems and frame monitoring efforts. Over the past two years, eDNA has risen to prominence as a leading candidate to become a high-resolution tool for detecting ecological communities' constituent species, and could meet many of these aims. Here, we have used one small fragment of mtDNA to detect vertebrate taxa in a large mesocosm, in the process highlighting both the potential and the pitfalls of eDNA analysis for community-wide assessment. While we find that sequence proportions recovered from a marine environmental sample are likely to be a function of taxon abundance, a number of potential biases may interfere with both presence/absence determinations and estimates of relative abundances. Multiple marker genes, increased spatiotemporal sampling and sequencing depths and a better understanding of DNA shedding rates will be necessary to more accurately profile community composition using eDNA. Despite the methodological challenges inherent in eDNA analysis, the results presented here are a step towards using eDNA samples to quantify taxa in ecological communities in the field. Meeting this larger goal would be of immediate policy and ecological relevance, providing a powerful tool for environmental monitoring and for addressing questions of fundamental ecological and evolutionary importance.

## Supporting Information

Figure S1 **The effect of 1-parameter constraints (genus proportions of DNA generated in the tank) on model parameters for proportions of source DNA in tank.**
(TIFF)

Figure S2 **The effect of 1-parameter constraints (source proportions of DNA in tank) on model parameters for genus-level proportions of DNA generated in tank.**
(TIFF)

Figure S3 **Sequence quality (Q score) by cycle (i.e., base position) for a representative set of sequences in the dataset.** Plotting function and analysis done using ShortRead library [3] of the Bioconductor package for R (R Core Team 2013). Gray-scale shading is proportional to the number of reads, with darkest shading (black) indicating the greatest number of reads. Orange lines are the median (solid) and 25^th^ and 75^th^ percentiles (dotted); light green line is the mean. Total read counts shown: 1.23×10^6^. Other MiSeq runs included in the dataset analyzed in the main text—including three technical replicates of tank surface water and single samples of incurrent and feed—were of similar size and quality.
(EPS)

Figure S4 **Vertebrate composition by lowest taxonomic rank for the (A) tank, (B) intake and (C) feed samples for the Open Sea tank.** Taxonomic identification was determined using a 106 bp 12 s mitochondrial DNA fragment.
(EPS)

Figure S5 **Agarose gel of PCR products from** ***Chelonia mydas*** **primer amplifications on 1-L Open Sea Tank samples.** Lane 1, 100 bp DNA ladder (size marker); Lane 2, synthetic plasmid containing partial mitochondrial control region for *C. mydas* (positive control); Lane 3, 1-L tank sample from Feb. 2013; Lane 4, 1-L tank sample from Oct. 2012; Lane 5, No template control. Amplicons for the tank samples were Sanger sequenced, trimmed and compared against the NCBI nonredundant nucleotide database with best match to *C. mydas* (NCBI GI: 399886525).
(EPS)

Figure S6 **Sequence alignment of the primer regions for the 12S rRNA vertebrate primers in species found in the Open Sea Tank.** Base pair mismatches are highlighted in red. GenBank GI accession numbers follow the genus name. Sequence data for the 12S target region was not available for the tank genera *Sarda* and *Naucrates*.
(EPS)

Text S1 **Details of the mixing model used to estimate the source contribution of DNA from the intake water, feed and tank-generated DNA to the Open Sea Tank.**
(DOCX)
