# Supplementary figures and images for: Using Environmental DNA to Census Marine Fishes in a Large Mesocosm
Source: PLoS One. 2014 Jan 15;9(1):e86175. doi: 10.1371/journal.pone.0086175 (PMC3893283; doi:10.1371/journal.pone.0086175)

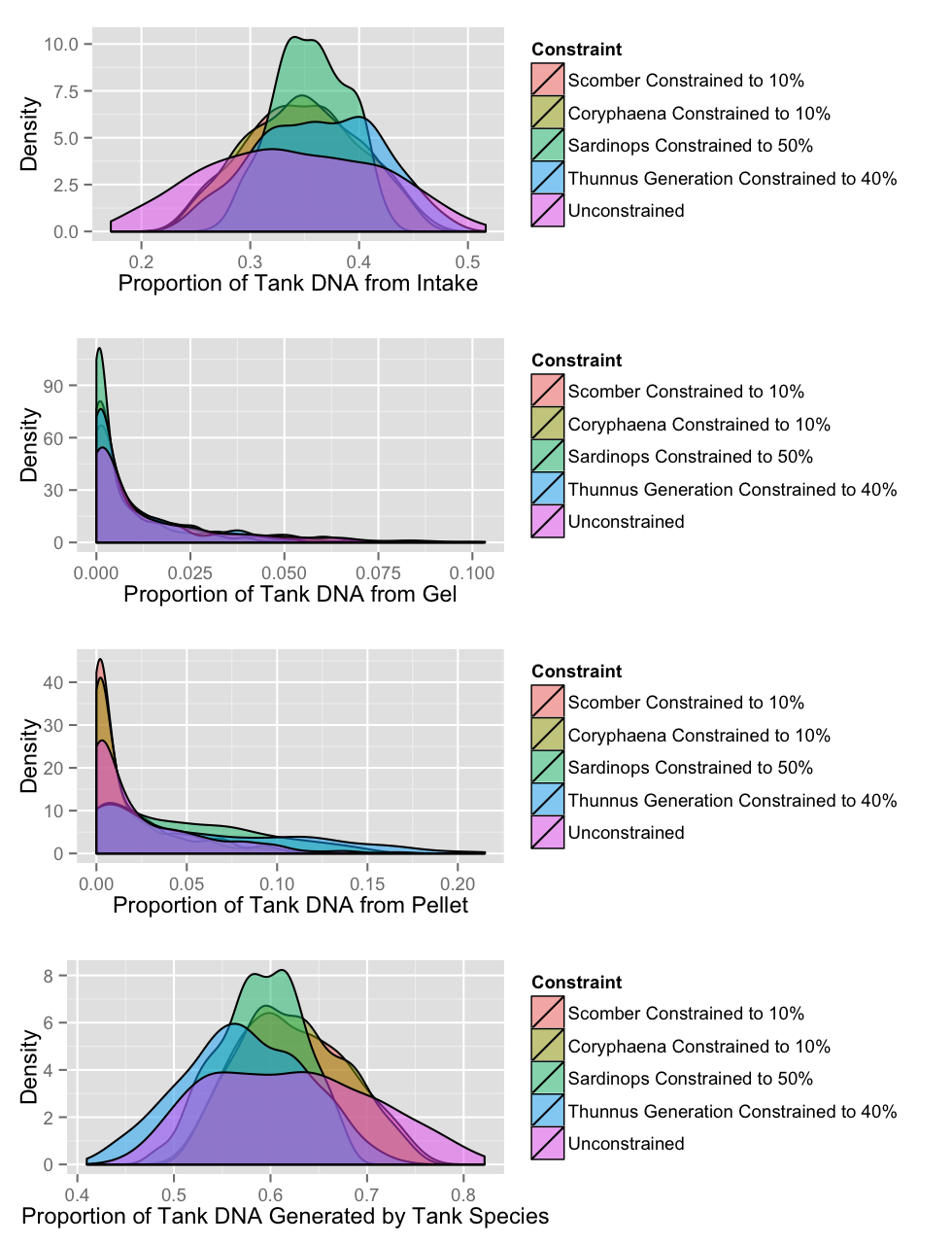

Supplement: Figure S1 — The effect of 1-parameter constraints (genus proportions of DNA generated in the tank) on model parameters for proportions of source DNA in tank. (TIFF) [file pone.0086175.s001.tiff]

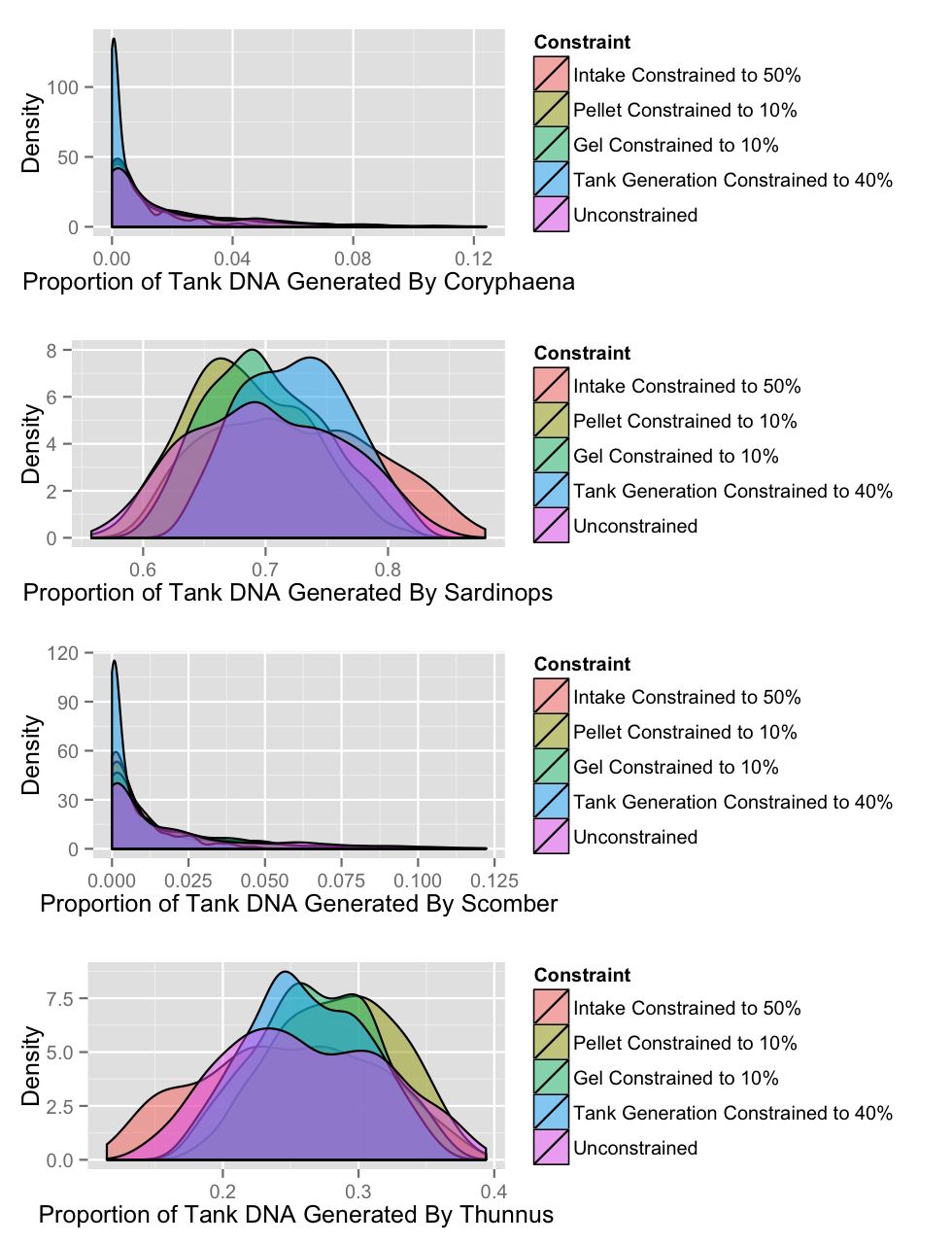

Supplement: Figure S2 — The effect of 1-parameter constraints (source proportions of DNA in tank) on model parameters for genus-level proportions of DNA generated in tank. (TIFF) [file pone.0086175.s002.tiff]
