# Supplementary material for: Using Environmental DNA to Census Marine Fishes in a Large Mesocosm
Source: PLoS One. 2014 Jan 15;9(1):e86175. doi: 10.1371/journal.pone.0086175 (PMC3893283; doi:10.1371/journal.pone.0086175)
Supplement: Text S1 — Details of the mixing model used to estimate the source contribution of DNA from the intake water, feed and tank-generated DNA to the Open Sea Tank. (DOCX) [file pone.0086175.s007.docx]

**Supplementary Methods**

**Mixing model**

As described in the main text, we developed a mixing model to estimate the contributions of four different sources of DNA in the sampled aquarium tank: intake, gel feed, pellet feed, and endogenously generated DNA from the tank species themselves. We estimated the values of eight unknowns (Eq. 1, main text) by sampling 5 million sets of values and evaluating the goodness-of-fit of the mixing model to the data in hand. These data consisted of sequence data from the intake, both types of feed, and three technical replicates from the surface water in the aquarium tank. After exploratory modeling, we generated final model iterations presented by sampling Dirichlet distributions for the parameter sets with alpha = 1 (intake and endogenously generated DNA sources, and *Sardinops* and *Thunnus* sequence proportions) or alpha = 0.3 (feed DNA sources and *Coryphaena* and *Scomber* sequence proportions). Results from this more restricted sampling scheme were similar to the exploratory scheme in which alpha = 1 for all parameter values.

The vast majority of sampled sets of values resulted in a poor fit between data and model, as was expected given the random sampling scheme covering most of the available parameter space. We therefore focused on models in the 99^th^ percentile of goodness-of-fit, yielding 5*10^4^ sets of parameter values (Figure 3). From the distributions of these parameter values, we developed confidence intervals for each from the central 95% of each distribution.

This optimization—which was complicated by the need to estimate both portions of the data and portions of the model—was robust to a suite of perturbations. We constrained each of the eight parameters in turn, assessing the effect of each on the distribution of the other modeled parameters with 5*10^4^ sets of randomly chosen values (Figures S1 and S2), noting little effect. Constraints shown are intended to stress the model by setting each parameter to a value outside its unconstrained optimal range. Density and plotting were done with ggplot2 [1] in R [2].

**Supplementary References**

1. Wickham H (2009) ggplot2: elegant graphics for data analysis. New York: Springer. 213 p.

2. R Core Team (2013) R: A language and environment for statistical computing. R Foundation for Statistical Computing, Vienna, Austria. <http://www.R-project.org/>.

3. Morgan M, Anders S, Lawrence M, Aboyoun P, Pagès H, et al. (2009) ShortRead: a Bioconductor package for input, quality assessment and exploration of high-throughput sequence data. Bioinformatics 25: 2607-2608.
